# Patterns of uptake of prostate-specific membrane antigen (PSMA)-targeted ^18^F-DCFPyL in peripheral ganglia

**DOI:** 10.1007/s12149-017-1201-4

**Published:** 2017-08-22

**Authors:** Rudolf A. Werner, Sara Sheikhbahaei, Krystyna M. Jones, Mehrbod S. Javadi, Lilja B. Solnes, Ashley E. Ross, Mohamad E. Allaf, Kenneth J. Pienta, Constantin Lapa, Andreas K. Buck, Takahiro Higuchi, Martin G. Pomper, Michael A. Gorin, Steven P. Rowe

**Affiliations:** 1 Division of Nuclear Medicine and Molecular Imaging, The Russell H. Morgan Department of Radiology and Radiological Science, Johns Hopkins University School of Medicine, 601 N. Caroline St., Baltimore, MD 21287 USA; 2 Department of Nuclear Medicine, University Hospital Würzburg, Würzburg, Germany; 3 Texas Urology Specialists, Dallas, TX USA; 4 The James Buchanan Brady Urological Institute and Department of Urology, Johns Hopkins University School of Medicine, Baltimore, MD USA; 5 Department of Bio Medical Imaging, National Cardiovascular and Cerebral Research Center, Osaka, Japan

**Keywords:** ^18^F-DCFPyL, Imaging pitfalls, Prostate cancer, PSMA, Ganglia

## Abstract

**Objective:**

Radiotracers targeting prostate-specific membrane antigen (PSMA) have increasingly been recognized as showing uptake in a number of normal structures, anatomic variants, and non-prostate-cancer pathologies. We aimed to explore the frequency and degree of uptake in peripheral ganglia in patients undergoing PET with the PSMA-targeted agent ^18^F-DCFPyL.

**Methods:**

A total of 98 patients who underwent ^18^F-DCFPyL PET/CT imaging were retrospectively analyzed. This included 76 men with prostate cancer (PCa) and 22 patients with renal cell carcinoma (RCC; 13 men, 9 women). Scans were evaluated for uptake in the cervical, stellate, celiac, lumbar and sacral ganglia. Maximum standardized uptake value corrected to body weight (SUV_max_), and maximum standardized uptake value corrected to lean body mass (SUL_max_) were recorded for all ganglia with visible uptake above background. Ganglia-to-background ratios were calculated by dividing the SUV_max_ and SUL_max_ values by the mean uptake in the ascending aorta (Aorta_mean_) and the right gluteus muscle (Gluteus_mean_).

**Results:**

Overall, 95 of 98 (96.9%) patients demonstrated uptake in at least one of the evaluated peripheral ganglia. With regard to the PCa cohort, the most frequent sites of radiotracer accumulation were lumbar ganglia (55/76, 72.4%), followed by the cervical ganglia (51/76, 67.1%). Bilateral uptake was found in the majority of cases [lumbar 44/55 (80%) and cervical 30/51 (58.8%)]. Additionally, discernible radiotracer uptake was recorded in 50/76 (65.8%) of the analyzed stellate ganglia and in 45/76 (59.2%) of the celiac ganglia, whereas only 5/76 (6.6%) of the sacral ganglia demonstrated ^18^F-DCFPyL accumulation. Similar findings were observed for patients with RCC, with the most frequent locations of radiotracer uptake in both the lumbar (20/22, 90.9%) and cervical ganglia (19/22, 86.4%). No laterality preference was found in mean PSMA-ligand uptake for either the PCa or RCC cohorts.

**Conclusion:**

As PSMA-targeted agents become more widely disseminated, the patterns of uptake in structures that are not directly relevant to patients’ cancers must be understood. This is the first systematic evaluation of the uptake of ^18^F-DCFPyL in ganglia demonstrating a general trend with a descending frequency of radiotracer accumulation in lumbar, cervical, stellate, celiac, and sacral ganglia. The underlying biology that leads to variability of PSMA-targeted radiotracers in peripheral ganglia is not currently understood, but may provide opportunities for future research.

## Introduction

Recently, PET radiotracers targeting prostate-specific membrane antigen (PSMA), labeled with either ^68^Ga or ^18^F, have demonstrated favorable results for imaging men with prostate cancer (PCa) in a variety of clinical contexts [1–3]. Moreover, the potential utility of imaging non-prostate malignancies such as renal cell carcinoma (RCC) has begun to be more widely reported [4]. Compared to the more commonly used ^68^Ga-labeled PSMA-targeted agents, the ^18^F-labeled PSMA-targeted radioligand ^18^F-DCFPyL possesses the inherent advantages of a longer physical half-life, lower positron energy, and higher positron yield, and there have also been suggestions of a higher detection rate for sites of disease as well as an increased tumor-to-background ratio [5].

Regarding the biodistribution of PSMA-targeted agents, the variability of ^18^F-DCFPyL uptake in several solid organs was recently assessed and demonstrated a visually homogenous uptake in the normal liver along with intrinsic variability even lower than 2-deoxy-2-[^18^F]fluoro-d-glucose (^18^F-FDG). Uptake was also noted in the lacrimal glands, salivary glands, spleen, kidneys, and small bowel [6]. ^68^Ga-PSMA-HBED-CC, the most commonly used of the ^68^Ga-labeled PSMA-targeted radiotracers, parallels the biodistribution of ^18^F-DCFPyL, with focal uptake in all the same organs and with both radiotracers having the highest uptake in the kidneys [6]. The similar biodistributions of these PSMA-targeted agents suggest that normal variant sites of uptake and potential interpretive pitfalls will be similar between these compounds. Indeed, a number of potential false-positive findings have been described that the interpreting imaging specialist should be aware of while analyzing ^18^F-DCFPyL or ^68^Ga-labeled PSMA-targeted PET scans, including increased radiotracer accumulation in various benign entities (e.g., in adrenal or thyroid adenomas as well as in Paget’s disease [7–9]).

Interestingly, PSMA expression has been described in nervous tissue, where this protein is known as glutamate carboxypeptidase II or *N*-acetylated-alpha-linked acidic dipeptidase (NAALADase). In fact, PSMA has been recognized as a potential therapeutic target in several neurological disorders [10, 11]. PSMA-targeted PET radiotracer uptake has also been seen in sympathetic chain and ganglion structures and should be considered physiologic rather than pathologic, particularly in the celiac and stellate ganglia [12]. To further investigate the pattern of PSMA-targeted radiotracer uptake within ganglia on a larger scale, we performed a qualitative and quantitative analysis of the peripheral ganglia of patients with PCa and RCC who were imaged with ^18^F-DCFPyL PET/CT on one of several prospective protocols evaluating this radiotracer. We would anticipate that the results of this study would be applicable to other PSMA-targeted radiotracers.

## Patient and methods

### Patient population

In total, 98 patients who underwent ^18^F-DCFPyL PET/CT imaging between March 2015 and October 2016 were included in this retrospective post hoc evaluation: 76 men with known PCa and 22 patients (9 women) with diagnosed RCC [4]. All patients were originally imaged as part of institutional review board-approved protocols prospectively evaluating ^18^F-DCFPyL PET/CT (ClinicalTrials.gov identifiers NCT02151760, NCT02523924, NCT02687139). All patients signed written informed consent and ^18^F-DCFPyL was used under the auspices of an FDA Investigational New Drug application (IND 121064).

### Imaging procedure

As per our standard practice, patients were asked to be *nil per os* (with the exception of water and medications) for at least 4 h prior to radiotracer injection. ^18^F-DCFPyL was synthesized as previously described [13]. Integrated PET/CT using either a Discovery RX 64-slice PET/CT (General Electric, Waukesha, WI, USA) or a Biograph mCT 128-slice PET/CT (Siemens, Erlangen, Germany) operating in 3D emission mode with CT attenuation correction was performed in all patients. ^18^F-DCFPyL ≤9 mCi (≤333 MBq) was administered intravenously and after an uptake time of approximately 60 min, acquisitions from the mid-thigh to the vertex of the skull were conducted, covering six to eight bed positions (depending on patient height and the scanner) with patients in the supine position.

### Imaging analysis

**Fig. 1:**
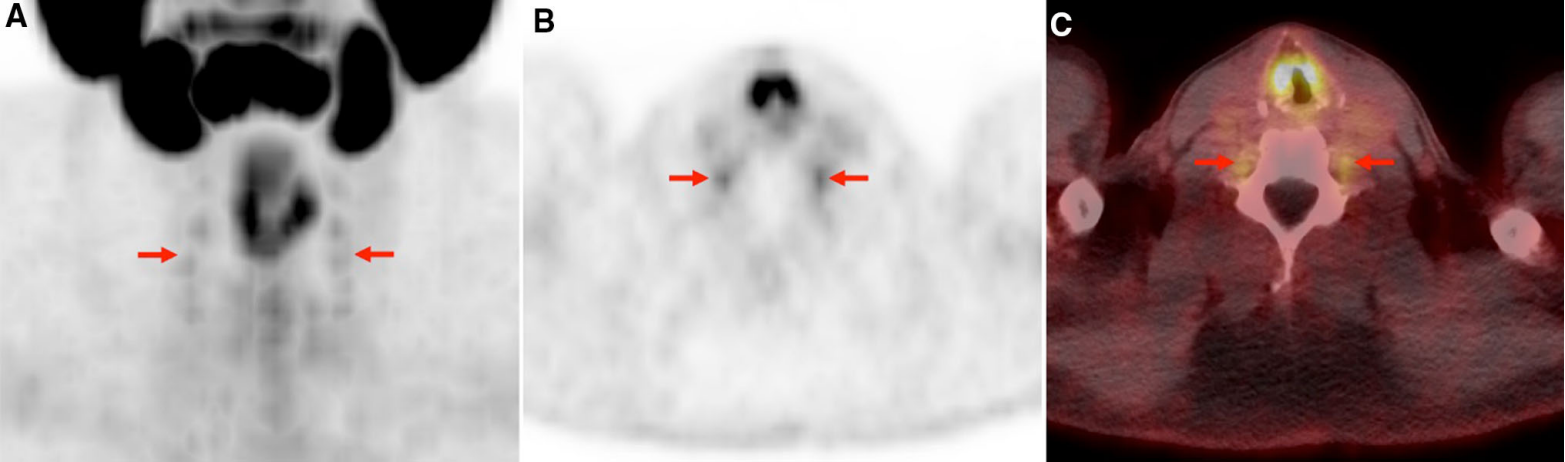
**a** Anterior view of ^18^F-DCFPyL maximum intensity projection (MIP) image from a 56-year-old PCa patient undergoing preoperative staging. Mild radiotracer uptake is seen in multiple cervical DRG bilaterally (*red arrows*). **b** Axial ^18^F-DCFPyL PET and **c** axial ^18^F-DCFPyL PET/CT fusion images from the C5–6 level also show mild radiotracer uptake in the C6 DRG bilaterally (*red arrows*). Note normal physiologic biodistribution of this radiotracer including uptake in the major salivary glands and structures of the larynx

**Fig. 2:**
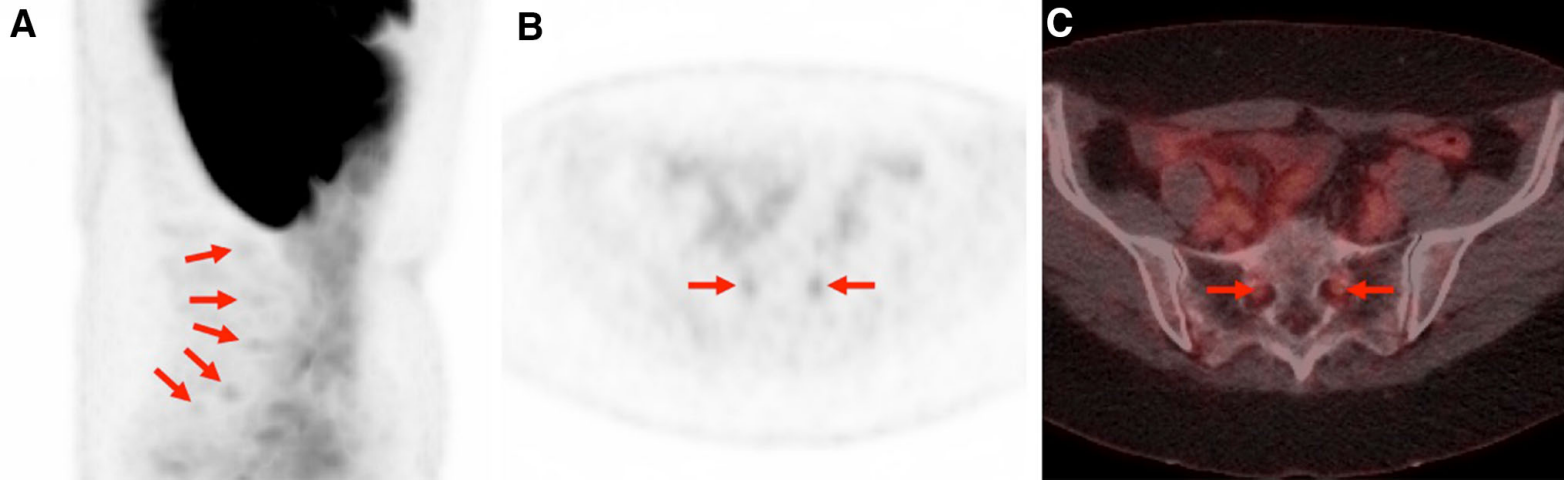
**a** Sagittal view of ^18^F-DCFPyL MIP image from a 58-year-old female patient with history of metastatic RCC and undergoing a staging examination. Multiple lumbar and sacral DRG show mild uptake (*red arrows*). **b** Axial ^18^F-DCFPyL PET and **c** axial ^18^F-DCFPyL PET/CT through the S1–2 level both demonstrate mild uptake of the S1 DRG (*red arrows*). Again, a portion of the normal physiologic biodistribution of the radiotracer is apparent including uptake in the patient’s remaining kidney, liver, and bowel

**Fig. 3:**
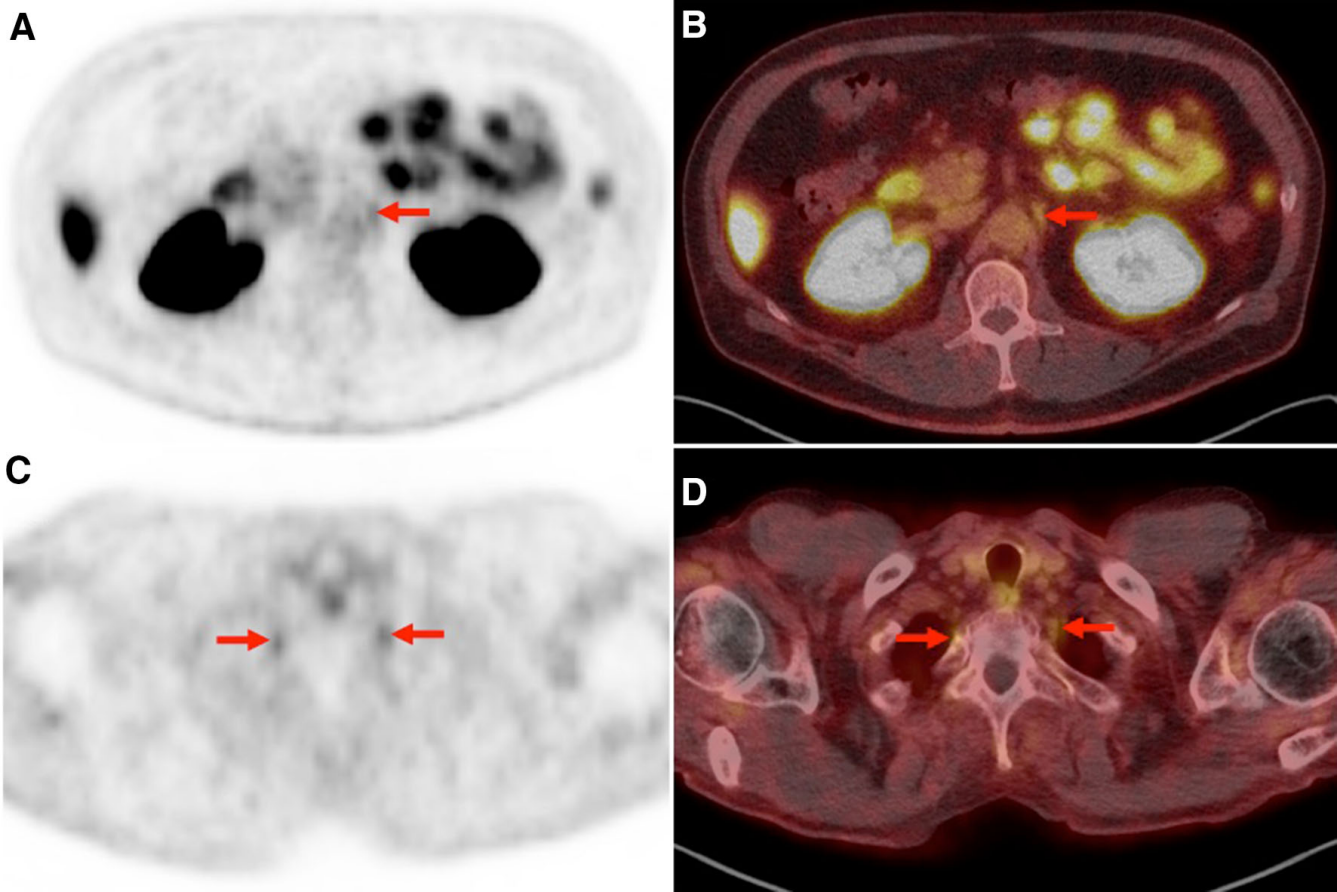
**a** Axial ^18^F-DCFPyL PET and **b** axial ^18^F-DCFPyL PET/CT images from a 66-year-old male patient undergoing staging evaluation for recurrent PCa. Focal mild-moderate radiotracer uptake is noted in the left celiac ganglion (*red arrow*). A similar degree of uptake was seen in the right celiac ganglion (not shown). **c** Axial ^18^F-DCFPyL PET and **d** axial ^18^F-DCFPyL PET/CT images from a 67-year-old male with recurrent PCa. Mild uptake is present in the stellate ganglia bilaterally (*red arrows*)

Imaging data were analyzed using a Syngo.via workstation (Siemens, Erlangen, Germany). PET, CT, and hybrid PET/CT imaging overlay could be assessed for viewing in all 98 patients. Two experienced nuclear medicine physicians (SPR and RAW) evaluated each PET for abnormal findings in the expected locations of the cervical, stellate, celiac, lumbar, and sacral dorsal root ganglia (DRG). Only those lesions that were visually determined as positive (i.e., those which demonstrated focally, discrete radiotracer retention matched to an area on CT in the expected location of a ganglion) were measured and included in the quantitative analysis. Examples of detected lesions are provided in Figs. 1, 2, 3.

To derive maximum standardized uptake value corrected to body weight (SUV_max_) and maximum standardized uptake value corrected to lean body mass (SUL_max_), a spherical volume of interest (VOI) was placed over each ganglion on the PET images. While at our institution, we prefer SUL_max_ for clinical ^18^F-FDG PET reads; our group has previously found that SUV_max_ is a simpler and equivalent semi-quantitative metric for ^18^F-DCFPyL PET [6]. Subsequently, the readers ruled out that that no other abnormal ^18^F-DCFPyL uptake findings could be detected in the volumes. A reference region was defined by drawing a VOI (diameter of 30 mm) involving the ascending aorta (Aorta_mean_) and the right gluteus muscle (Gluteus_mean_) to derive ganglia-to-aorta as well as ganglia-to-muscle ratios.

### Statistical analysis

Data are presented as mean ± standard deviation (SD) for continuous variables and frequency and percentage for categorical variables. Analysis was performed on a patient-, side- (left/right) and ganglia-level basis. To determine the significance of differences between two groups, the independent Student’s *t* test and paired t test (left vs. right side analysis) were performed. A two-tailed *p* value of less than 0.05 was considered statistically significant. Statistical analysis was performed using IBM SPSS Statistics (v.22, Chicago, IL, USA).

## Results

A total of 76 PCa patients with a mean age of 64 ± 7.2 years (range 47–81 years) and 22 RCC patients with a mean age of 57.8 ± 10.1 (range 34–73 years) were included in our analysis. The majority of the subjects were of white race for both the PCa (*n* = 65, 85.5%) and the RCC (*n* = 21, 95.5%) cohort. The clinical indication for imaging included preoperative PCa staging (18/76, 23.7%) and evaluation of recurrent/metastatic disease (58/76, 76.3%). For the RCC patients, similar indications were noted with preoperative staging in 6/22 (27.3%) and recurrent/metastatic disease evaluation in 16/22 (72.7%).

### All patients

In 95/98 (96.9%) patients, visually discernible PSMA-targeted ligand uptake in at least one of the above-indicated peripheral ganglia were identified (96.0% of PCa and 100.0% RCC patients). PSMA-ligand uptake above background was found in 70/98 (71.4%) patients in cervical ganglia, 60/98 (61.2%) patients in stellate ganglia, 57/98 (58.2%) patients in celiac ganglia, 75/98 (76.5%) patients in lumbar ganglia, and 8/98 (8.2%) patients in sacral ganglia. The mean SUV_max_ ± SD were 1.82 ± 0.34, 1.67 ± 0.47, 1.77 ± 0.59, 1.76 ± 0.31, and 1.91 ± 0.45 in the cervical, stellate, celiac, lumbar and sacral ganglia, respectively.

No laterality preference was found in ^18^F-DCFPyL uptake in cervical, stellate, celiac, lumbar and sacral ganglia when comparing the left and right SUV values, neither for the PCa nor for the RCC cohort (*p* > 0.05 for all comparisons).

Table 1 summarizes the frequency of ^18^F-DCFPyL uptake and the involved peripheral ganglia in both the PCa and RCC cohorts. The mean SUV_max_, SUL_max_ and the background ratios (Aorta_mean_, Gluteus_mean_) of the detected ganglia are given in Table 2. The displayed uptake values are generally quite low; however, the low blood pool and background activity of ^18^F-DCFPyL enable a robust differentiation of ganglia from surrounding tissue [3].

**Table 1:** Characteristics of peripheral ganglia identified on ^18^F-DCFPyL PET in patients with prostate cancer (*n* = 76) and renal cell carcinoma (*n* = 22)

Peripheral ganglia	No. of positive patients	No. of positive patients with bilateral involvement	No. involved ganglia
Prostate cancer			
Cervical	51/76 (67.1%)	30/51 (58.8%)	102 R, 86 L
C3	16/76	11/16	14 R, 13 L
C4	22/76	10/22	18 R, 14 L
C5	20/76	11/20	19 R, 12 L
C6	29/76	14/29	23 R, 20 L
C7	20/76	11/20	14 R, 17 L
C8	19/76	5/19	14 R, 10 L
Stellate	50/76 (65.8%)	20/50 (40.0%)	41 R, 29 L
Celiac	45/76 (59.2%)	30/45 (66.7%)	39 R, 36 L
Lumbar	55/76 (72.4%)	44/55 (80.0%)	111 R, 103 L
L2	13/76	8/13	10 R, 11 L
L3	38/76	29/38	34 R, 33 L
L4	46/76	30/46	40 R, 36 L
L5	28/76	22/28	27 R, 23 L
Sacral	5/76 (6.6%)	2/5 (40.0%)	4 R, 3 L
Renal cell carcinoma			
Cervical	19/22 (86.4%)	15/19 (78.9%)	44 R, 48 L
C3	12/22	9/12	10 R, 11 L
C4	5/22	2/5	2 R, 5 L
C5	10/22	5/10	7 R, 8 L
C6	9/22	4/9	6 R, 7 L
C7	12/22	9/12	10 R, 11 L
C8	10/22	5/10	9 R, 6 L
Stellate	10/22 (45.4%)	7/10 (70.0%)	8 R, 9 L
Celiac	12/22 (54.5%)	1/12 (8.3%)	5 R, 8 L
Lumbar	20/22 (90.9%)	17/20 (85.0%)	43 R, 44 L
L2	7/22	4/7	6 R, 5 L
L3	15/22	12/15	13 R, 14 L
L4	17/22	11/17	13 R, 15 L
L5	13/22	8/13	11 R, 10 L
Sacral	3/22 (13.6%)	1/3 (33.3%)	1 R, 3 L

*R* right, *L* left

**Table 2:** Quantitative analysis of the investigated peripheral ganglia identified on ^18^F-DCFPyL PET for both cohorts

Peripheral ganglia	SUV_max_	SUL_max_	SUV_max_/Gluteus_mean_	SUL_max_/Gluteus_mean_	SUV_max_/Aorta_mean_	SUL_max_/Aorta_mean_
Prostate cancer						
Cervical	1.82 (0.36)	1.36 (0.26)	4.37 (1.30)	4.16 (1.21)	1.36 (0.39)	1.49 (1.00)
Stellate	1.69 (0.49)	1.28 (0.37)	4.23 (1.48)	3.93 (1.31)	1.30 (0.45)	1.45 (1.05)
Celiac	1.69 (0.58)	1.28 (0.46)	4.29 (2.11)	4.11 (1.70)	1.26 (0.57)	1.43 (1.43)
Lumbar	1.78 (0.31)	1.34 (0.22)	4.55 (1.14)	4.26 (1.34)	1.35 (0.36)	1.47 (0.74)
Sacral	1.85 (0.52)	1.33 (0.30)	4.20 (1.97)	3.85 (1.16)	1.37 (0.39)	1.37 (0.39)
Renal cell carcinoma						
Cervical	1.81 (0.29)	1.30 (0.22)	3.73 (1.33)	3.67 (1.39)	1.48 (0.91)	1.60 (1.13)
Stellate	1.56 (0.36)	1.17 (0.36)	3.45 (1.90)	3.35 (2.00)	1.06 (0.29)	1.08 (0.30)
Celiac	2.06 (0.55)	1.44 (0.42)	4.30 (1.61)	3.90 (1.84)	2.18 (2.14)	2.09 (2.03)
Lumbar	1.71 (0.32)	1.21 (0.17)	3.43 (1.13)	3.43 (1.21)	1.43 (0.88)	1.52 (1.05)
Sacral	2.03 (0.38)	1.42 (0.38)	4.30 (2.59)	4.29 (2.63)	3.73 (2.26)	3.64 (2.14)

Mean and standard deviation are given

### Uptake in ganglia of PCa patients

With regard to the cervical ganglia, 51/76 (67.1%) PCa patients had uptake in at least one cervical ganglia with similar findings for both sides [left 86 ganglia, right 102 ganglia, with bilateral uptake in at least one ganglion in 30/51 (58.8%) patients]. The most frequent site of radiotracer accumulation was observed at the C6 level with 29/76 (38.2%) patients having positive uptake. Regarding the stellate ganglia, 50/76 (65.8%) were positive [left 29 ganglia, right 41 ganglia, and bilateral 20/50 (40.0%)], whereas for celiac ganglia, 45/76 (59.2%) showed ^18^F-DCFPyL uptake [left 36 ganglia, right 39 ganglia, and bilateral 30/45 (66.7%)]. Of note, the lumbar ganglia were positive in the majority of the subjects (55/76, 72.4%) with closely analogous findings for both sides [left 103 ganglia, right 111 ganglia, and bilateral 44/55 (80.0%)]. Discernible radiotracer uptake was most commonly observed at the L4 level with 46/76 (60.5%) positive subjects. Regarding sacral ganglia, only 5/76 (6.6%) demonstrated ^18^F-DCFPyL accumulation [left 3 ganglia, right 4 ganglia, and bilateral 2/5 (40%)].

### Uptake in ganglia of RCC patients

Radiotracer uptake was observed in the cervical ganglia in 19/22 patients with RCC [86.4%; left 48 ganglia, right 44 ganglia, and bilateral 15/19 (78.9%)]. Most frequently uptake could be found in the level of C3 and C7 in 12/22 (54.5%), respectively. In the stellate ganglia, 10/22 (45.5%) of the patients demonstrated discernible radiotracer uptake and in the celiac ganglia, 12/22 (54.5%) subjects were positive. Of note, the most frequent site of uptake was in the lumbar ganglia [20/22 (90.9%), left 44 ganglia, right 43 ganglia and bilateral, 17/20 (85%)] with most frequent findings in L4 [17/22 (77.3%)]. Only 3/22 (13.6%) of the RCC patients demonstrated ^18^F-DCFPyL accumulation in sacral ganglia.

## Discussion

The present study describes the uptake patterns in peripheral ganglia, which can act as potential pitfalls when clinically interpreting ^18^F-DCFPyL PET/CT scans for both PCa and RCC. There was a general trend with a descending frequency of radiotracer accumulation in lumbar, cervical, stellate, celiac, and sacral ganglia. An inexperienced clinical reader could identify uptake in these physiological structures as potential sites of metastatic disease, although many of the peripheral ganglia that have been observed to have uptake are somewhat separated anatomically from typical lymph nodes that become involved with metastatic disease (the most clear exception being the celiac ganglia). Additionally, the cervical, lumbar, and sacral ganglia could potentially be mistaken for bone metastatic disease, particularly if there is slight misregistration between the PET and CT acquisitions or if the uptake is distinctly unilateral.

Generally, the stellate ganglion can also be easily identified on anatomical imaging and this was a common site of uptake in our study (65.8% in PCa) [14]. The ganglia most likely to be misinterpreted as disease-involved lymph nodes are the celiac, as described in a manuscript with extensive pathologic correlation by Krohn and coworkers who were utilizing the PSMA-targeted agent ^68^Ga-PMSA-HBED [12]. Recently, using the same radiotracer, Kanthan et al. reported similar results, with up to 81% positive uptake findings in the celiac and 74% in the stellate ganglia [15]. In the present study, we evaluated the PSMA-targeted ligand uptake across all peripheral ganglia, both in RCC and PCa patients using ^18^F-DCFPyL. Compared to the above-mentioned investigations, our number of positive findings was lower in the celiac (59.2%) and stellate ganglia (65.8%). This may be due to a variety of factors including potential improved spatial/contrast resolution of an ^18^F-labeled radiotracer [16] or perhaps the different underlying chemical structures of the radiotracers which could profoundly impact localization in a primarily hydrophobic environment such as a ganglion [5, 17]. Moreover, the study population of our cohort was almost 10 years younger than the populations in the studies by Krohn [12] and Kanthan [15]. In a study of more than 7400 participants, Steiber and coworkers concluded that the average physical health in an elder population declines age-dependently [18]. Keeping in mind that physical inactivity is an important cause of most chronic diseases as well as playing a role in pain perception [19], our “younger” population included in the current study might have suffered from less biological chronic disease/pain-associated sensation of the celiac ganglia.

Investigating lumbar DRG by 3D magnetic resonance imaging in healthy volunteers, 90% of the DRG showed an intermediate signal intensity, which is in line with the frequency of uptake in our PCa (72.4%) and RCC (90.9%) cohorts [20]. Hence, while interpreting ^18^F-DCFPyL PET, a clinical reader should keep in mind that physiological uptake in the lumbar region is a frequent phenomenon which could be falsely mistaken as a metastatic site, similar to potential false-positive findings in the celiac ganglia [12]. The same tendency of gradually increasing findings of DRG from L1 to L5 [entire cohort: L2, 20/98 (20.4%) vs. L4, 63/98 (64.3%)], the more frequent occurrence of bilateral ganglia in L4 (entire cohort: 41/63, 65.1%) as well as the fact that the right-left difference in PSMA-uptake reached no significance, could also be observed in the present study [20, 21].

This study has several limitations. First, given its retrospective nature, further confirmatory studies are warranted. Second, no histologic proof of the obtained PET results can be given in light of the benign nature and characteristic locations of ganglia. Moreover, ganglia uptake may be underestimated as partial volume effects have to be considered in small structures less than 15 mm.

## Conclusion

Clinical readers should be conscious of potential false-positive findings while interpreting PSMA-targeted PET, and uptake in the lumbar, cervical, stellate and celiac ganglia could be potentially misinterpreted as metastatic lesions. We provide a description of the patterns of ^18^F-DCFPyL uptake in peripheral ganglia in a large and varied patient cohort. Future research should aim to define the mechanisms underlying the variability of PSMA-targeted radiotracer uptake by peripheral ganglia.
